# Near-Infrared Triggered Degradation for Transient Electronics

**DOI:** 10.1021/acsomega.3c07203

**Published:** 2024-01-04

**Authors:** Emin Istif, Mohsin Ali, Elif Yaren Ozuaciksoz, Yagız Morova, Levent Beker

**Affiliations:** † Department of Molecular Biology and Genetics, Faculty of Engineering and Natural Science, Kadir Has University, Istanbul 34083, Turkey; ‡ Department of Biomedical Sciences and Engineering, Koç University, Rumelifeneri Yolu, Sarıyer, Istanbul 34450, Turkey; § Koç University Surface Science and Technology Center (KUYTAM), Rumelifeneri, Istanbul 34450, Turkey; ∥ Department of Mechanical Engineering, Koç University, Rumelifeneri Yolu, Sarıyer, Istanbul 34450, Turkey; ⊥ Nanofabrication and Nanocharacterization Centre for Scientific and Technological Advanced Research, Koç University, Rumelifeneri Yolu, Sarıyer, Istanbul 34450, Turkey

## Abstract

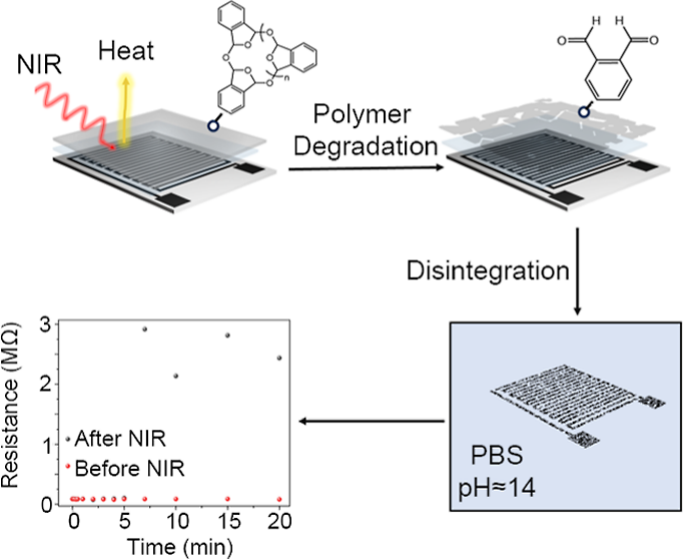

Electronics that disintegrate after stable operation present exciting opportunities for niche medical implant and consumer electronics applications. The disintegration of these devices can be initiated due to their medium conditions or triggered by external stimuli, which enables on-demand transition. An external stimulation method that can penetrate deep inside the body could revolutionize the use of transient electronics as implantable medical devices (IMDs), eliminating the need for secondary surgery to remove the IMDs. We report near-infrared (NIR) light-triggered transition of metastable cyclic poly(phthalaldehyde) (cPPA) polymers. The transition of the encapsulation layer is achieved through the conversion of NIR light to heat, facilitated by bioresorbable metals, such as molybdenum (Mo). We reported a rapid degradation of cPPA encapsulation layer about 1 min, and the rate of degradation can be controlled by laser power and exposure time. This study offers a new approach for light triggerable transient electronics for IMDs due to the deep penetration depth of NIR light through to organs and tissues.

## Introduction

Disintegrable materials possess a potential interest for fabrication of transient electronic devices, which represent a new class of technology for biomedical implantable devices,^1–3^ temporal memory devices,^4–6^ environmental^7^ and soil sensor^8^ applications. Transient electronic devices can disintegrate fully or partially in a predetermined time or by using external stimuli to trigger disintegration. Therefore, material engineering is crucial for developing this technology and its use in various applications. For instance, such devices can be revolutionary in implantable medical devices (IMDs) by providing complete disintegration of the device after stable operation or a predetermined time in the body environment. On the other hand, because conventional medical devices are optimized for long-term operation, secondary surgery becomes inevitable in case of removal of the device.^9^

In current practice, the dissolution of the substrate or encapsulation layer, which protects the electronic circuits built on a substrate, usually controls the operation time of transient devices.^9,10^ However, significant efforts have been made to control the dissolution time in biofluids or aqueous solutions by varying the thickness or crystallinity of polymer materials.^11^ Another applicable method is tuning the chemical properties of biodegradable polymers, such as polycaprolactone, poly(glycolic acid), poly(lactic acid), poly(lactic-*co*-glycolic acid) to determine the degradation time by a synthetic approach such as varying the molecular weight of polymers or changing copolymer ratios.^12,13^ Although these approaches are effective for developing transient electronics, they provide a predetermined device lifetime tuned by material selection and do not allow for an on-demand device transition. As a result, devices begin to deteriorate once implanted, and device characteristics start to vary immediately.

Recently, stimuli-responsive materials have attracted the attention of researchers interested in the fabrication of transient electronic devices that degrade in response to a specific stimulus such as light,^15–17^ thermal,^14,15^ electrochemical,^16^ and chemical.^17–19^ As a result, polymers that depolymerize rapidly under mild conditions can become suitable candidates for the encapsulation layer to realize the on-demand transition. cyclic poly(phthalaldehyde) (cPPA) which is a metastable polymer with low ceiling temperatures (*T*_c_), is a promising material for both encapsulation and substrate layer of transient electronic devices that can offer precise control over the lifetime of the transient electronics due to its rapid depolymerization upon backbone bond cleavage by acid or heat stimuli.^20–23^

Hernandez et al.^24^ demonstrated a phototriggerable transient electronic device fabricated on a cPPA substrate with a photoacid generator (PAG). The results show that rapid degradation begins once the device is exposed to UV light due to the released hydrochloric acid (HCl) from PAG under UV exposure, which can destroy both cPPA and magnesium (Mg) electrodes. Although the UV-triggering approach yields promising results for transient electronics, it has limited applications in bioelectronic applications, such as IMDs, due to the low penetration depth of UV light through the human skin.^25^

Park et al.^15^ reported a thermally triggered transient electronic device that included a wax encapsulation layer, cPPA substrate, and Mg electrode. In the study, they encapsulated acid microdroplets in wax, and melting of the wax released the encapsulated acid, allowing acidic degradation of cPPA and Mg. The study also demonstrated that inductive coupling can be used to control heat triggering at a distance. Despite the study’s unique approach, degradation of the device occurs around 55 °C, limiting the device’s integration for IMD applications because skin and tissue can be damaged at that temperature.

Recently research has also demonstrated moisture-triggered degradation of transient electronics. Gao et al.^26^ synthesized a moisture-sensitive polyanhydride-based material that can be hydrolyzed in the presence of moisture, with the polymer degradation producing organic acids that cause the degradation of inorganic electronic materials and components. The humidity level and the monomer composition of the polyanhydride polymer can be used to control the device’s transience lifetime. Although the presented method is suitable for many applications, such as transistors, optoelectronics, and diodes, the high water content in body fluids limits its bioelectronic application, particularly for IMDs.

We present a new approach for triggered transient electronics in which the encapsulation layer is made of cPPA and is destroyed by near-infrared (NIR) light triggering, which can overcome the potential challenges of light-triggered transient processes for IMDs due to NIR light’s higher penetration depth to the skin than UV light. The cPPA transition is carried out by exposing cPPA-coated Mo circular-shaped specimens and electrodes to 976 nm NIR light. The thermal depolymerization of cPPA layers caused by Mo’s conversion of NIR light to heat is attributed to the cPPA transition. The cPPA layer’s transience was tuned by the laser source’s power and irradiation time, which can cause partial or complete degradation of cPPA. Furthermore, we demonstrate the transience of the cPPA layer by coating a Mo-based resistor and interdigitated electrode (IDE) that resembles a typical IMD sensor. The results show that cPPA degradation reduces capacitance by partially removing cPPA as the dielectric layer. Furthermore, once the cPPA encapsulation layer is destroyed, the resistor’s performance in the presence of a basic solution is altered because the Mo electrode rapidly dissolves in a basic solution. By providing NIR-triggered degradation, this approach provides a novel approach for various types of transient electronics, particularly IMDs.

## Result and Discussion

**Figure 1:**
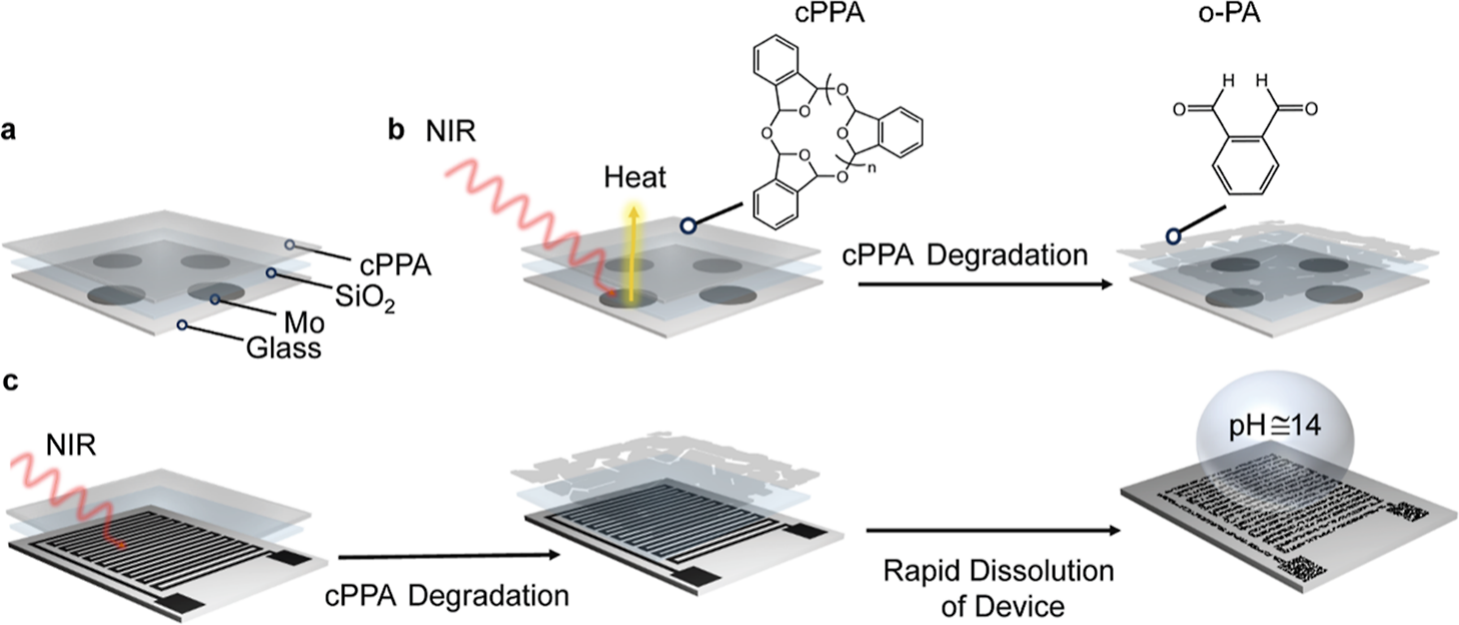
NIR triggered the transience of cPPA. (a) Mo-based sample device structure, (b) conversion of NIR to heat by Mo metal triggers the degradation of cPPA, and (c) illustration of transient electronic device that can disintegrate by NIR triggering and rapid dissolution of the device.

The proposed transition method enables degradation of the cPPA encapsulation layer via a NIR light trigger, which can be used for electronic devices with on-demand transience. Figure 1 illustrates the sample device patterns, layers, and NIR light triggerable approach, where metal patterns convert light to heat, leading to cPPA depolymerization. Once the encapsulation layer disintegrates by NIR light triggering, the bioresorbable metal-based device components can rapidly dissolve in the basic medium. In this study, molybdenum (Mo) metal was employed due to its biocompatibility and bioresorbable properties. Molybdenum is an essential element for the human body, crucial for various biological functions, with a recommended daily intake ranging from 0.05 to 400 mg per day.^18^ Moreover, Mo can undergo dissolution under physiological pH conditions, forming its metabolizable form, the molybdate anion (MoO_4_^2–^). MoO_4_^2–^ can be rapidly regulated within the body and excreted through urine, typically at levels between 10 and 16 μg/L.^27^ The degradation of Mo in pH 7 and sodium chloride (NaCl) solution is reported to occur at rates between 10^–4^ and 10^–3^ μm h^–1^ at room temperature and 0.00083 μm h^–1^ in phosphate-buffered saline (PBS) solution (pH 7.4) at 37 °C for 10 μm thick Mo foil.^28^

To implement the proposed method, first, cPPA was synthesized as reported in the literature^24,29^ by employing cationic polymerization of *o*-phtalaldehdyde (*o*-PA) as monomer and borontrifluoride diethyl etherate (BF_3_·OEt_2_) as the initiator (Figure S1, Supporting Information). The polymer was synthesized at −78 °C to prevent depolymerization of cPPA in acidic conditions. Structural characterization of synthesized cPPA was conducted by using nuclear magnetic resonance (NMR) and Fourier transform infrared (FTIR) spectroscopy techniques. ^1^H NMR spectrum of cPPA was compared with the monomer (*o*-PA) ^1^H NMR spectrum (Figure S2, Supporting Information). cPPA spectrum shows broad peaks at 7.5 ppm, assigned to the aromatic structure and acetal groups at 6.5 ppm. Since both peaks are absent in the *o*-PA structure, cPPA formation was confirmed by the NMR spectrum. Further, FTIR characterization was performed for both cPPA and *o*-PA (Figure S3, Supporting Information). FTIR spectra confirm the successful synthesis of cPPA due to the disappearance of carbonyl (C=O) peaks of *o*-PA at 1750 cm^–1^ and the emergence of acetal (C–O–C) peaks around 1100 cm^–1^ after polymerization.

Once the polymer structure was confirmed, cPPA films were prepared on a Mo-coated glass substrate (1 cm × 1 cm) to expose the cPPA to NIR light. The polymer solution was prepared in the concentration of 100 mg/mL in chloroform and 15 μL plasticizer [di(propylene glycol) dibenzoate] were added due to the low flexibility of cPPA. Further, cPPA was coated on a Mo-coated glass substrate using a spin coater to expose the polymer film to NIR light. A cPPA-coated substrate was placed in a sample holder located around 20 cm from the NIR light source (Figure S4, Supporting Information). The fiber-coupled diode laser (BWT-K976AA2VN-18W) was collimated with a 3 cm lens, and a 0.4 cm spot size was obtained. Collimated beam was further focused with another 15 cm converging lens. The sample was placed between the focal point and the converging lens, where the spot size coincided with the structure size. The laser spot, which has a 0.4 cm spot size, was visualized using R-Scope Infrared Viewer, and the laser was spotted in the middle of the substrate. Six samples were prepared and exposed to 1 W NIR light for different periods. Mo’s conversion of NIR into heat was evident even through physical observation, as the substrate could be felt warmer by touching it with fingers.

**Figure 2:**
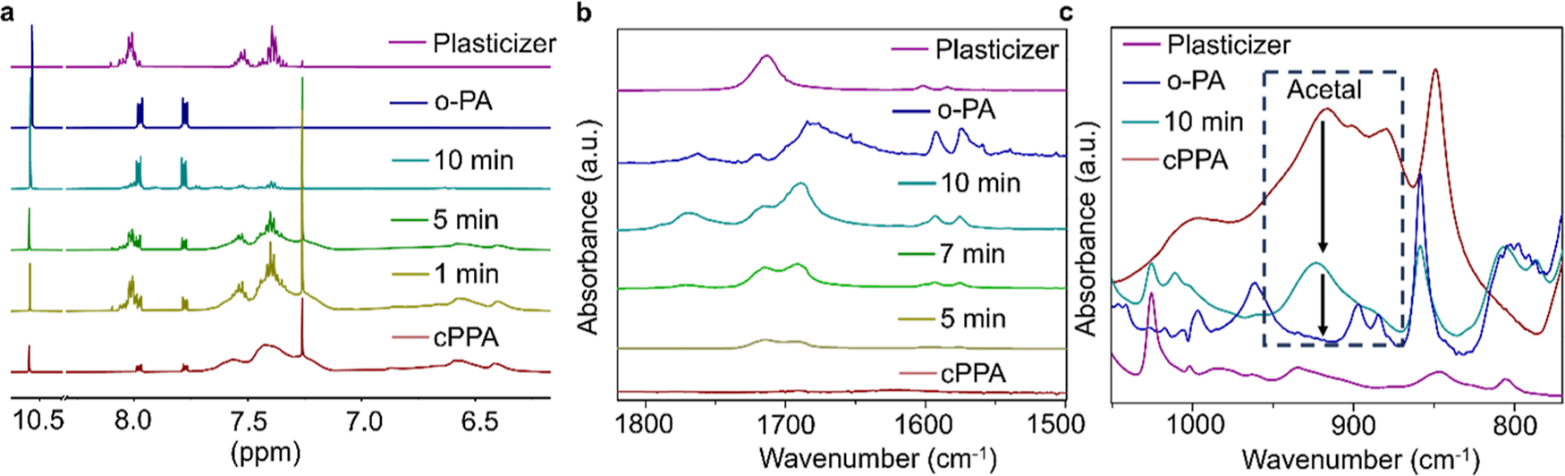
Structural characterization of NIR-exposed and nonexposed cPPA, monomer, and plasticizer samples. (a) ^1^H NMR characterization of the samples, (b) FTIR characterization of degraded and nondegraded polymers carbonyl peaks at 1800–1500 cm^–1^, and (c) acetal backbone peaks at 1100–800 cm^–1^.

After samples were exposed to NIR, chemical changes in polymer structure were characterized using NMR and FTIR analysis. cPPA samples were dissolved from Mo substrate using CDCl_3,_ and the solutions were analyzed using proton nuclear magnetic spectroscopy (^1^H NMR). ^1^H NMR spectra were taken for six samples exposed to 1 W NIR for 1–10 min. Before irradiation ^1^H NMR spectra of cPPA show resonances of cPPA as broad peaks at 7.5 ppm (aromatic) and 6.5 ppm (acetal groups) and traces of *o*-PA. After exposure of samples to NIR, starting from 1 min, the broad peak of the cPPA, both aromatic and acetals, no longer existed, and the intensity of the aldehyde peak at 10.5 ppm started to increase. Further, some plasticizer traces emerged as sharp peaks in the ^1^H NMR spectra (Figure 2a). ^1^H NMR analysis showed that by increasing time, the degradation of cPPA results in the formation of *o*-PA as the primary product. ^1^H NMR spectra of the samples from 11 to 0 ppm were given in Figure S5, Supporting Information.

FTIR spectroscopy was used as a complementary technique to monitor the chemical changes in the cPPA structure after NIR exposure (Figure 2b,c). FTIR spectra were taken from NIR-exposed samples and gently peeled off of the Mo substrate by using a flat spatula. cPPA FTIR spectrum can be distinguished from the *o*-PA spectra due to acetal backbone peaks at 900–1100 cm^–1^. After NIR exposure, the intensity of the acetal peaks decreases, and carbonyl peaks around 1750 cm^–1^ corresponding to reversion to *o*-PA appear due to depolymerization of cPPA. Although the *o*-PA spectrum shows only one intense peak in the carbonyl region around 1700 cm^–1^ and a slight peak around 1800 cm^–1^, the intensity at 1800 cm^–1^ increased once the cPPA fully depolymerized after 10 min. The second carbonyl peak around 1800 cm^–1^ is due to the oxidation of aromatic aldehydes in air.^23,24^ FTIR spectra of the samples in the range of 3500–600 cm^–1^ were given in Figure S6, Supporting Information.

**Figure 3:**
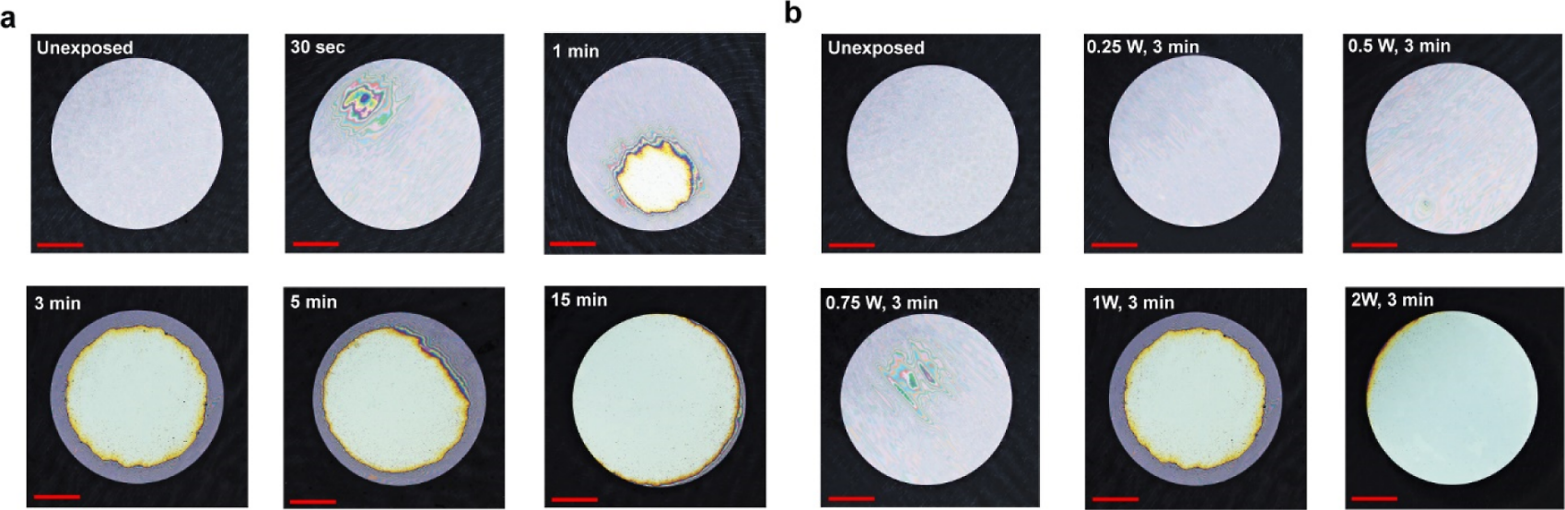
Visualization of cPPA degradation on Mo after NIR exposure. Confocal microscopy images of NIR-exposed cPPA- coated Mo samples, (a) effect of different time exposure and (b) effect of different laser powers. The scale bar of the images is 500 μm.

Next, the physical deformation of the cPPA due to chemical degradation by NIR was characterized using confocal and optical microscopy, profilometry, and micromechanical testing. For microscopy studies, we designed and fabricated Mo-based circle shape specimens (around 2 mm diameter each) on a glass substrate (Figure S7, Supporting Information) using e-beam evaporation. After Mo was deposited on a glass substrate, a thin SiO_2_ layer was coated to protect Mo from organic solvents during spin coating. SiO_2_ is widely used in the literature as bioresorbable material for transient electronics.^6,18,19^ Although SiO_2_ can be used as a thermal insulator, the thermal conductivity of the SiO_2_ depends on the fabrication process, thickness, and temperature.^30–32^ In our study, we did not observe significant thermal insulation properties of SiO_2_ using the chemical vapor deposition method to obtain a 150 nm thick layer of SiO_2_. Each circle was irradiated on various time scales, and the deformation of spin-coated films of cPPA was observed. Confocal microscopy images show that the degradation of cPPA starts after 30 s of irradiation using 1 W of NIR laser power. After 3 min of irradiation, more than 50% of cPPA was degraded, and complete degradation was observed in 15 min of irradiated samples (Figure 3a). The degradation of cPPA on irradiated Mo circles was also observable by using an optical microscope (Figure S8, Supporting Information). For optical microscopy images, the samples were tilted to arrange the contrast for having observable degradation. The results indicate that cPPA depolymerization occurs immediately once the NIR light is converted to heat by Mo metal.

Furthermore, we analyze the effect of laser power on degradation by irradiating the samples for 3 min using various laser powers such as 250, 500, 750, and 2 W. Although the degradation of cPPA was observable using 750 mW due to the reflection of the light from cPPA, the degraded area was smaller than the samples, which were irradiated with 1 W for 3 min (Figure 3b). On the other hand, using 2 W of laser power, we observed complete degradation of cPPA-coated area. The degraded area of the 2 W irradiated sample is almost two times bigger than the degraded area of the 1 W irradiated sample (Figure 3). The microscopy image results show that NIR exposure to cPPA-coated Mo leads to the degradation of cPPA, and the amount of degradation and degradation rate depends on the time of exposure and laser power. While a higher NIR laser power may be necessary for biomedical implantable devices to penetrate deeper into the skin,^33^ the proposed NIR-based approach can be readily integrated for implantable device applications through power modulation.

Once the cPPA degradation is confirmed by the NIR method, to gain a deeper understanding of temperature dependency on cPPA degradation, the degradation of cPPA was induced through thermal processing and subsequently examined the ^1^H NMR spectrum. The cPPA polymer films on Mo sample were prepared by spin coating and the samples were heated to around 35, 70–75, and 90–100 °C. After the thermal treatment, the cPPA samples were dissolved in CDCl_3_ and ^1^H NMR characterizations were conducted (Figure S9, Supporting Information). The results showed that the complete depolymerization of cPPA takes place around 70–75 °C. This result is in accordance with the literature,^15^ which shows the degradation of cPPA around 55 °C. The treatment around 90–100 °C, shows that depolymerization occurs and the aldehyde peaks around 10.5 ppm disappear, which can be related with transformation of aldehydes of *o*-PA monomer to carboxylic acid group. Based on the NMR results, the NIR triggering method may cause a local temperature increase around 70–75 °C to trigger the complete degradation on Mo samples.

To gain better insight into the cPPA degradation, the thickness of cPPA was characterized using a profilometer. The thickness of cPPA (∼3 μm) was analyzed by scanning the exposed circle shape Mo and the nonexposed substrate area to compare the cPPA thickness. The profilometer results were obtained from different time-irradiated samples using 1 W laser power. The results indicate that once the profilometer tip reaches to cPPA degraded part on the Mo circle, the thickness of the film decreases by about 1 μm for 3 min of irradiated samples and 2 μm for 5 and 10 min of irradiated samples due to degradation of cPPA placed on NIR-exposed Mo circles (Figure S10, Supporting Information).

Surface characterization of the cPPA-coated Mo circles was further analyzed using contact angle measurements. Since cPPA is a hydrophobic polymer, we hypothesize that the degradation of cPPA can disrupt the hydrophobicity of the cPPA, and the contact angle with water may vary after degradation. Therefore, we compared the contact angles of bare Mo, spin-coated films of cPPA on Mo, and irradiated cPPA-coated films on Mo. The contact angle of bare Mo and cPPA resulted in 60.88° and 93.77°^,^ respectively (Figure S11, Supporting Information). The cPPA-coated sample exhibits above 90°, which is related with hydrophobicity of cPPA as reported.^34,35^ After exposing cPPA-coated Mo circle to NIR (1 W for 5 min), the contact angle was reduced to 75.24°, indicating that the hydrophobicity of cPPA was disrupted due to cPPA film degradation.

**Figure 4:**
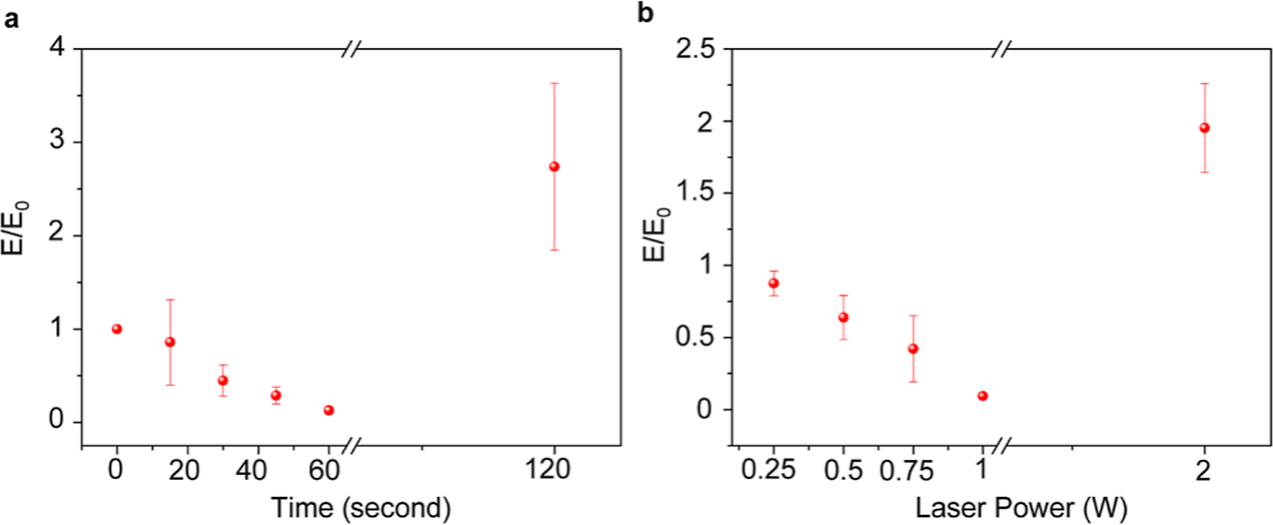
Mechanical characterization of NIR-exposed cPPA-coated Mo. Elastic modulus of cPPA films coated on Mo as a function of, (a) exposure time (1 W for 15, 30, 45, 60, and 120 s) and (b) laser power (0.25, 0.5, 0.75, 1, and 2 W for 120 s). The error bars for *E*/*E*_0_ measurements indicate standard deviations, which were obtained by analysis of the cPPA film at least three times (*N* = 3) from different regions. The data are presented as mean values ± s.d.

Encapsulation layers are critical to the mechanical integrity of transient electronic devices. Therefore, to characterize the mechanical integrity of the cPPA as an encapsulation layer, dynamic mechanical testing was performed to analyze the physical degradation of the cPPA film coated on Mo. We quantified the elastic modulus of spin-coated films of cPPA while they were exposed to NIR at various laser powers (0.25, 0.5, 0.75, 1, and 2 W for 120 s) and time (1 W for 15, 30, 45, 60, and 120 s) to determine the time scale of physical degradation (Figure 4a,b). The cPPA films were tested until NIR exposure caused the cPPA to degrade. There was almost no change in the elastic modulus when the specimen was exposed to the NIR light at 0.25 W for 2 min. The loss in elastic modulus was observed when both parameters (power and time) were increased. The decrease in the elastic modulus after NIR exposure can be attributed to the chemical degradation of cPPA, which leads to the physical deformation of cPPA films. Once cPPA degradation begins, the polymer film becomes more softer due to the plasticizer content and leads to decrease in elastic modulus (Figure 4). When the cPPA thin film (∼3 μm) was exposed for 2 min to 2 W or 1 W for 120 s, the exposed cPPA was fully degraded, and mechanical measurement provided elastic modulus of underneath layers such as SiO_2_ and Mo. Considerable degradation in elastic modulus is noticed prior to the film’s failure (Figure 4).

To demonstrate the application of the cPPA film as an encapsulation layer for biodegradable electronic devices, Mo-based IDEs and a resistor were fabricated and encapsulated with cPPA (Figure S12, Supporting Information). The fabrication of Mo-based electrodes on a glass substrate was achieved by electron beam (e-beam) deposition. On Mo electrodes, a SiO_2_ layer was deposited to protect the Mo electrodes during the spin-coating process of cPPA. Once the cPPA was coated on electrodes, the electrodes were exposed to 1 W of NIR for 10 min to ensure the degradation of the encapsulation layer. The hole-shaped deformation on cPPA films after NIR exposure was observed by optical microscopy (Figure S13, Supporting Information). Next, we aimed to characterize the capacitance and resistance of the corresponding electrodes.

**Figure 5:**
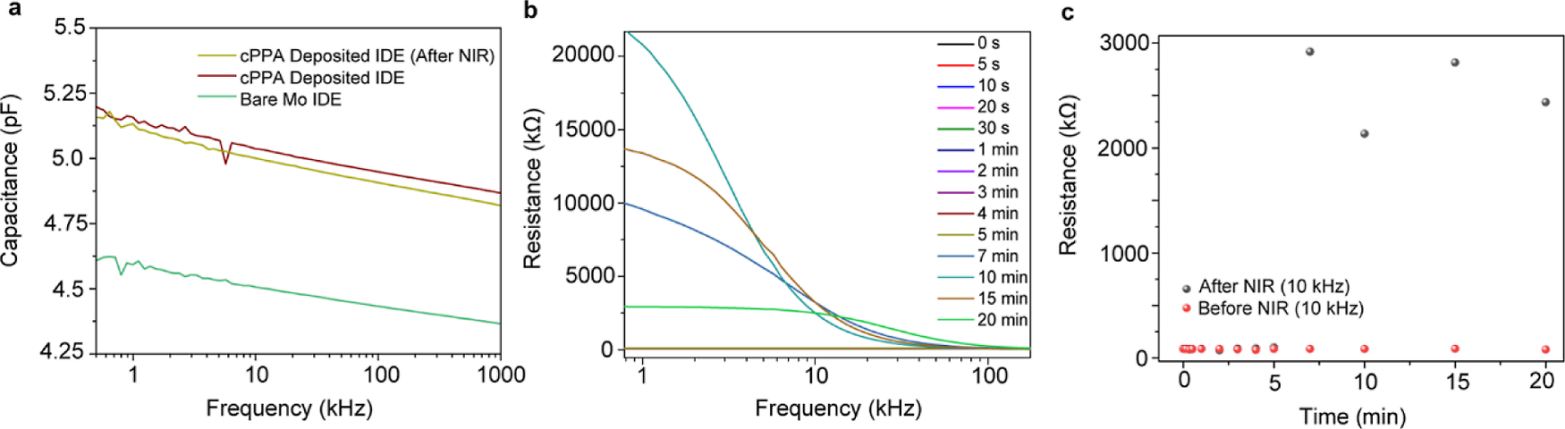
Demonstration of NIR triggered degradation of the cPPA encapsulation layer as transient electronics. (a) The capacitance data of cPPA-coated Mo interdigitated electrode before and after NIR exposure. (b) The resistivity change data of NIR-exposed cPPA- coated Mo resistors after basic PBS treatment (the data from 0 to 4 min given in Figure S16, Supporting Information). (c) The resistivity change data of NIR-exposed cPPA-coated Mo resistors at 10 kHz as a function of time after basic PBS treatment (the data from 0 to 5 min given in Figure S17, Supporting Information).

First, capacitance measurements were conducted on IDEs using an impedance analyzer. Once the bare electrode was coated with cPPA, the capacitance of the electrodes was increased since cPPA behaves as a dielectric layer over the electrodes according to the following formula
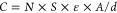
where *N* is the number of IDEs, *S* is a geometrical factor, ε is the permittivity of cPPA and SiO_2_, and *A* and *d* are the area and gap of the IDE, respectively. After exposure of the IDEs to NIR, the electrode’s capacitance decreases (Figure 5) due to the degradation of the dielectric layer, as the dielectric layer does not cover the entire electrode surface due to the degradation of the cPPA film. While the capacitance change is minimal because of the very small degraded area of cPPA, the repeatability of the capacitance change was confirmed by conducting the same experiment with three different electrodes (Figure S15 in the Supporting Information).

Finally, a resistive electrode was also characterized to observe the resistance change. The electrode was exposed to 1 W NIR for 10 min to induce the cPPA degradation. Once the physical deformation occurred on the cPPA encapsulation layer, approximately 25 μL of basic 1× PBS (pH 14) was dropped on the degraded area of cPPA film, and the basic solution was left on the degraded area starting from 5 s to 20 min. We aimed at the rapid degradation of Mo electrodes by using a basic PBS solution. After incubation in basic PBS, the electrode was rinsed with (deionized) DI water and dried with air before resistance measurements. We observed that the basic solution triggers the degradation of Mo and SiO_2_ by leaking through the degraded cPPA encapsulation (Figure S14, Supporting Information). Therefore, the resistance of the electrode increases since the conductive connections were lost (Figure 5c). A similar experiment was conducted by using an unexposed sample. The unexposed sample treated with basic PBS shows an increase in resistivity after 10 min (Figure 5c). However, the resistivity increase was lower than that in the NIR-exposed sample. The slight change in resistivity in the unexposed sample can be due to the partial detachment of the cPPA layer during the PBS incubation.

## Conclusions

The material and electrode systems presented here offer a novel approach to producing NIR light-triggerable transient electronics. The transience of the cPPA encapsulation layer relies on the NIR light-to-heat conversion by metal electrodes. The transience of the cPPA layer is fast and tunable by laser power or exposure time, which provides a programmable on-demand transition of electronic devices. Here, Mo-based patterns and electrical components prove the methodology of the NIR triggerable degradation, but it is important to note that the approach is not limited to Mo electrodes and various types of metals such as Zn, Fe, or Mg can be used since they may convert NIR light to heat. The presented approach was demonstrated on capacitive and resistive electrodes to provide insight into transient electronics applications. Once the partial degradation of the cPPA encapsulation layer is triggered by NIR light, complete disintegration of the bioresorbable metals is conducted in the basic solution. Our NIR-based triggerable transition shows great promise for transient electronics in the IMD field since it offers an on-demand transition with NIR light, which provides deep penetration through the organs and tissues.

## Experimental Section

### Materials

*o*-PA ≥ 97% (HPLC), dichloromethane (CH_2_Cl_2_) (>99% purity), borontrifluoride diethyl etherate (BF_3_·OEt_2_) (for synthesis), CDCl_3_ (99.8 at. % D), 3 Å molecular sieve, methanol (>99% purity) were received from Sigma-Aldrich. Glass substrates were received from Marienfeld with approximate thickness of 1 mm.

### Synthesis of cPPA

cPPA was synthesized according to a literature procedure.^24,29^ First, *ortho*-phthalaldehyde (*o*-PA) (2.00 g, 29.8 mmol) is weighed into a flame-dried Schlenk flask and dissolved in dry dichloromethane (16 mL). The solution is cooled to −78 °C using liquid nitrogen and an ethanol bath. Next, boron trifluoride etherate (0.04 mL, 0.325 mmol) was added dropwise through the septum via syringe. The reaction is left stirring at −78 °C for 2 h, and then pyridine (0.10 mL, 1.25 mmol) is added. The mixture is left stirring for 2 h at −78 °C and then brought to room temperature. The polymer was collected from the reaction mixture by precipitation, using methanol. The reaction mixture was poured dropwise into 250 mL of methanol with magnetic stirring. The white product is collected by filtration and then further purified by washing it with excess of methanol. Lastly, the polymer was dissolved in dicholoromethane and reprecipitated from methanol (1.40 g, 70%). ^1^H NMR and FTIR characterization of cPPA were provided in Supporting Information. Dry dichloromethane was prepared by adding 50 mL of dichloromethane over 10 g of freshly dried 3 Å molecular sieve in a dry Schlenk flask. Nitrogen was purged for 15 min from dichloromethane, and the flask was sealed and left under nitrogen overnight. Dry molecular sieves were dried under a vacuum at 200 °C for 24 h.

### Spin Coating of cPPA on Substrates

For the cPPA spin-coating process, first, the cPPA was dissolved in chloroform in 100 mg/mL concentration, and 15 μL of plasticizer (di(propylene glycol) dibenzoate) was added. Next, the cPPA solution dropped on 2.5 cm × 2.5 cm glass chips and spin coated at 1000 rpm for 10 s followed by 500 rpm for 10 s to get ∼3 μm thick film. The same parameters were used to coat the interdigitated and resistive electrodes.

### Fabrication of Mo Circular Pattern and Electrodes

A standard glass slide was cut into chips measuring 2.5 cm × 2.5 cm. The glass chips were cleaned with acetone and iso-propyl alcohol. The chips were then coated with HMDS (VP8 Vapor Primer, Suss MicroTec) at a temperature of 120 °C for 2 min.

The circular, resistor, and interdigitated Mo-based patterns were defined through UV lithography using the μMLA 100 system from Heidelberg Instruments, after the spin coating of AZ5214 positive resist provided by Microchemicals. The development process involved the utilization of AZ 726 MIF developer (Microchemicals) for a duration of 60 s. To remove excess resist from the developed regions, an oxygen-plasma treatment was conducted for 60 s using the SI 500 system from Sentech Instruments. Following the patterning step, a 50 nm thick layer of Mo was deposited through e-beam evaporation using the PVD 200 PRO-Line equipment from Kurt J. Lesker. The desired metal pattern was achieved by performing a lift-off process in acetone and followed by the deposition of 150 nm thick layer of SiO_2_ using plasma enhanced chemical vapor deposition.

### Capacitance and Resistance Measurement of Mo Electrodes

The capacitance changes of the Mo-based interdigitated electrodes were characterized using a Zurich Instruments MFIA Impedance Analyzer. The experiments were performed in sweeper mode in the frequency range of between 100 Hz and 5 MHz. The bare electrode and spin-coated cPPA electrodes were characterized before and after NIR exposure. For NIR-exposed sample 1 W of NIR power for 10 min was used to induce the cPPA degradation. The electrodes were connected to an impedance analyzer from the pad of the electrodes via flat alligator clips. The capacitance measurements were performed in air.

For the resistive measurements, the electrodes were coated with cPPA using a coated spin coater. The resistivity of the electrodes were characterized using a Zurich Instruments MFIA Impedance Analyzer in sweeper mode in the frequency range of between 100 Hz and 5 MHz. For resistivity measurement the NIR-exposed samples were treated with basic PBS solution from 5 s to 20 min to induce the degradation of the Mo-based electrodes. Basic PBS solution can immerse through the degraded areas that were created during NIR exposure and induce the rapid degradation of resistive electrodes. After each basic PBS treatment, the electrode was rinsed with DI water and dried before the resistivity measurements.

### NMR and FTIR Characterization of Bare cPPA and NIR- Exposed Samples

We conducted ^1^H NMR characterization of cPPA on a 400 MHz Bruker Avance Spectrometer. For sample preparation, 20 mg of cPPA was dissolved in 0.5 mL of CDCl_3_ and the result was analyzed using MestReNova (version 14.1.0-24037) software.

For the NIR degraded cPPA sample, 100 mg/mL cPPA solution in CHCl_3_ was drop casted on a Mo-coated glass substrate (1 cm × 1 cm). Then, the samples were exposed to NIR in different time periods using 1 W of power. Once the exposure was completed, the cPPA samples on glass-Mo substrate were dissolved using CDCl_3_ and ^1^H NMR analysis was performed.

For FTIR characterization, after NIR exposure, the sample was collected from the glass-Mo substrate by gently peeling using a flat spatula. It should be noted that for bare cPPA FTIR samples, the plasticizers were not used since the carbonyl group of plasticizers can interfere with the monomer peaks. FTIR analysis were performed using ThermoScientific iS20 FTIR, operating in attenuated total reflectance mode. FTIR spectra data were analyzed by using Thermo Fischer OMNIC Series Software.

It should be noted that SiO_2_ deposition was not employed for the glass-Mo substrates for NMR and FTIR analysis.

### Mechanical Characterization

For mechanical characterization, 2 sets of samples were prepared. The Mo circle shape specimens were fabricated and coated with cPPA by a spin-coating process. Six of the circles were exposed to NIR for 15, 30, 45, 60, and 120 s using 1 W of laser power. In another glass chip, 5 different Mo circle shape specimens were exposed to NIR for 120 s using 0.25, 0.5, 0.75, 1, and 2 W laser power. Once the samples were prepared, mechanical measurements were conducted under ambient conditions using the FT-MTA03 micromechanical assembly and testing system (FemtoTools AG, Zurich, Switzerland), configured with the compression method. A glass spherical tip indenter (FT-S200) with a diameter of 25 μm, offering a measurement range of ±200 μN and a resolution of 0.0005 μN, was employed to acquire force–displacement curves. The force–displacement data was utilized to calculate the elastic modulus. *E*/*E*_0_ measurements were repeated three times using different areas of the samples. A Detailed description of force–displacement curve analysis is given in Supporting Information.

### Microscopy Images

Optical images of the samples were taken using a Studio microscope Leica DM1000 (Leica Microsystems) and confocal microscop (LEXT OLS5100).

### NIR Setup

Our NIR setup for sample exposure consisted of a fiber-coupled laser diode (BWT-K976AA2VN-18W) having the central wavelength of 976 nm, a converging lens (*f* = 3 cm), a sample holder, and a power meter. The distance between the laser and the sample holder was about 20 cm. The NIR laser spot on the sample was visualized using R-Scope Infrared Viewer (FJW, P770, Optical System, USA). The detailed description of the setup is given in Figure S4, Supporting Information with images. The spot size of the collimated laser was also determined as 0.4 cm.

## Supporting Information Available

Synthesis method of cPPA, structural characterization of cPPA, NIR laser setup, structural characterization of NIR-exposed samples, optical images of exposed and nonexposed samples, profilometry characterization of the samples, contact angle characterizations, images of Mo-based electrodes (PDF)

## Abbreviations

cPPA: cyclic poly(phthalaldehyde)
IMDs: implantable medical devices
NIR: near-infrared
Mo: molybdenum
NMR: nuclear magnetic resonance
FTIR: Fourier-transform infrared
CHCl_3_: chloroform
*o*-PA: *o*-phtalaldehdyde
PBS: phosphate-buffered saline
DI: deionized, SiO_2_, silicon dioxide
PLA: polylactide
PLGA: poly lactic-*co*-glycolic acid
