# Supplementary material for: Near-Infrared Triggered Degradation for Transient Electronics
Source: ACS Omega. 2024 Jan 4;9(2):2528–35. doi: 10.1021/acsomega.3c07203 (PMC10795112; doi:10.1021/acsomega.3c07203)
Supplement: Supplementary file 1 — ao3c07203_si_001.pdf [file ao3c07203_si_001.pdf]

Supplementary Information

Near-Infrared Triggered Degradation for Transient Electronics

Emin Istif[*1], Mohsin Ali[2], Elif Yaren Ozuaciksoz[2], Yagız Morova[3], Levent Beker[*4,5]*

[1] Department of Molecular Biology and Genetics, Faculty of Engineering and Natural Science, Kadir Has University, Istanbul, 34083, Turkey

[2] Department of Biomedical Sciences and Engineering, Koç University, Rumelifeneri Yolu, Sarıyer, Istanbul, 34450, Turkey

[3] Koç University Surface Science and Technology Center (KUYTAM), Rumelifeneri, Istanbul, 34450, Turkey

[4] Department of Mechanical Engineering, Koç University, Rumelifeneri Yolu, Sarıyer, Istanbul, 34450, Turkey

[5]Nanofabrication and Nanocharacterization Centre for Scientific and Technological Advanced Research, Koç University, Rumelifeneri Yolu, Sarıyer, Istanbul, 34450, Turkey

Correspondence: emin.istif@khas.edu.tr, lbeker@ku.edu.tr

Table of Content

1. Synthesis of cPPA
2. Structural characterization of cPPA
3. NIR laser setup
4. Structural characterization of NIR exposed samples
5. Optical images of exposed and non-exposed samples
6. ^{1}H NMR characterization of thermally degraded cPPA film
7. Profilometry characterization of the samples
8. Contact angle characterizations
9. Mo-based electrodes
10. Capacitance data of Mo based capacitors
11. Resistance data of Mo based resistors
12. Mechanical analysis of the samples

1. **Synthesis of cPPA**

cPPA was synthesized according to a literature procedure[24,27]. Firstly, ortho-phthalaldehyde (o-PA) (2.00 g, 29.8 mmol) is weighed into a flame dried Schlenk flask and dissolved in dry dichloromethane (16 mL). The solution is cooled to -78 ˚C using liquid nitrogen and ethanol

bath. Next, boron trifluoride etherate (0.04 mL, 0.325 mmol) was added dropwise through the septum via syringe. The reaction is left stirring at -78 ˚C for 2 h, then pyridine (0.10 mL, 1.25 mmol) is added. The mixture is left stirring 2 h at -78 ˚C, then brought to room temperature. The polymer was collected from the reaction mixture by precipitation using methanol. The reaction mixture was poured dropwise into 250 mL of methanol under magnetic stirring. The white product is collected by filtration, then further purified by washing it with excess of methanol. Lastly, the polymer was dissolved in dicholormethane and re-precipitated from methanol. (1.40 g, 70%).^{1}H NMR and FTIR characterization of cPPA were provided in Supplementary Information. Dry dichloromethane was prepared by adding 50 ml of dichloromethane over 10 g of freshly dried 3A molecular sieve in a dry Schlenk flask. Nitrogen was purged 15 min from dichloromethane, and the flask was sealed and left under nitrogen overnight. Dry molecular sieves were dried under a vacuum at 200°C for 24 hours.

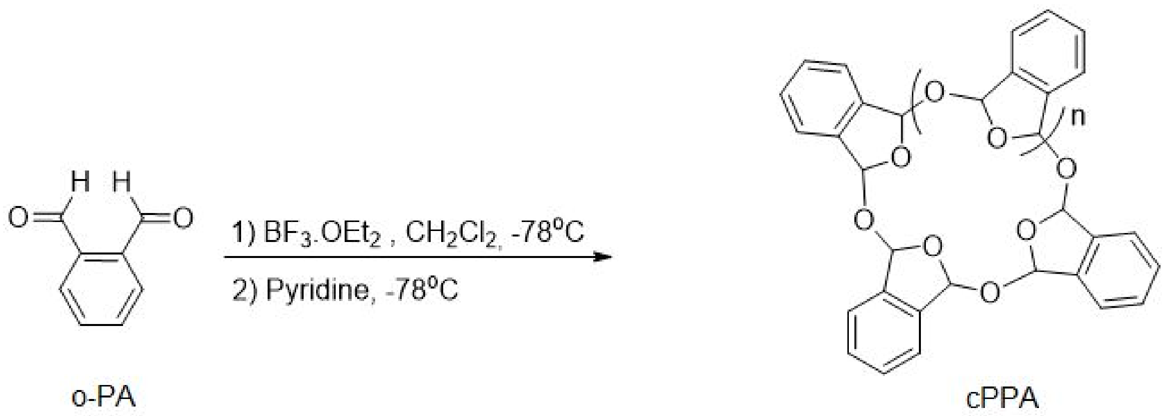

Figure S1. Synthesis of cPPA

2. Structural characterization of cPPA

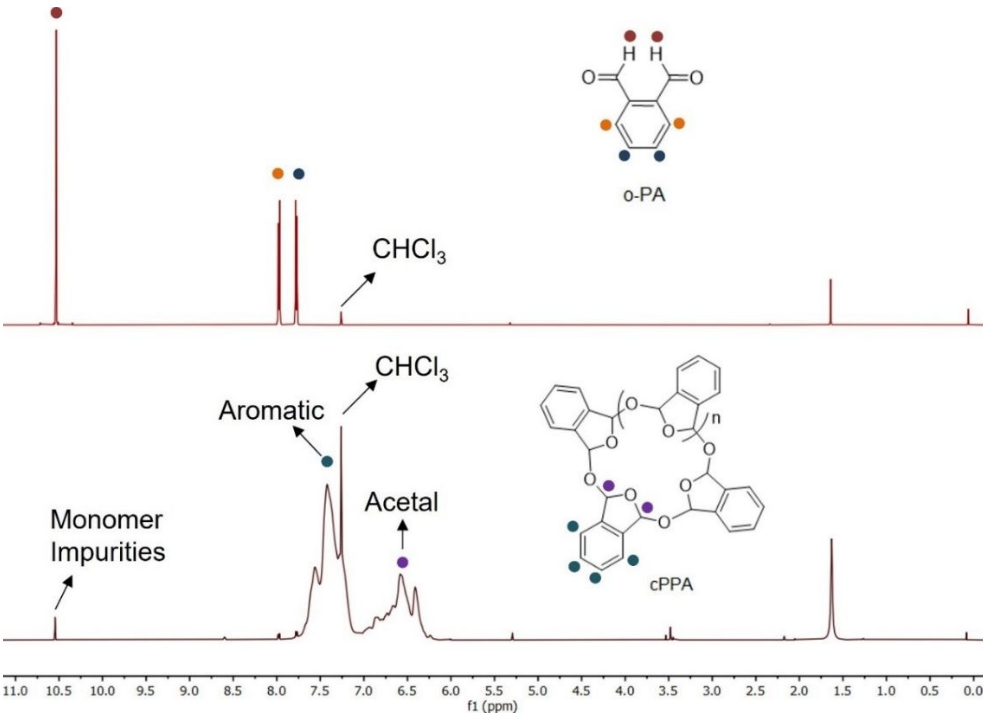

Figure S2. ^{1}H NMR spectra of o-PA (top) and cPPA (bottom) in $CDCl_3$

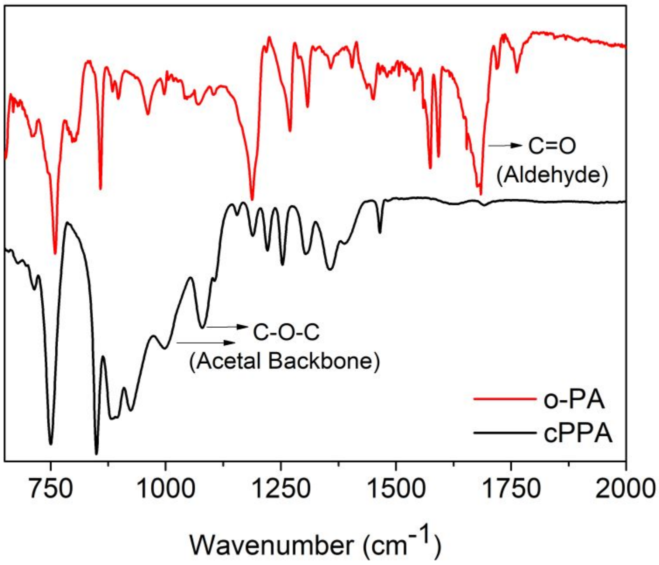

Figure S3. FTIR spectra of o-PA (red) and cPPA (black)

3. NIR Setup

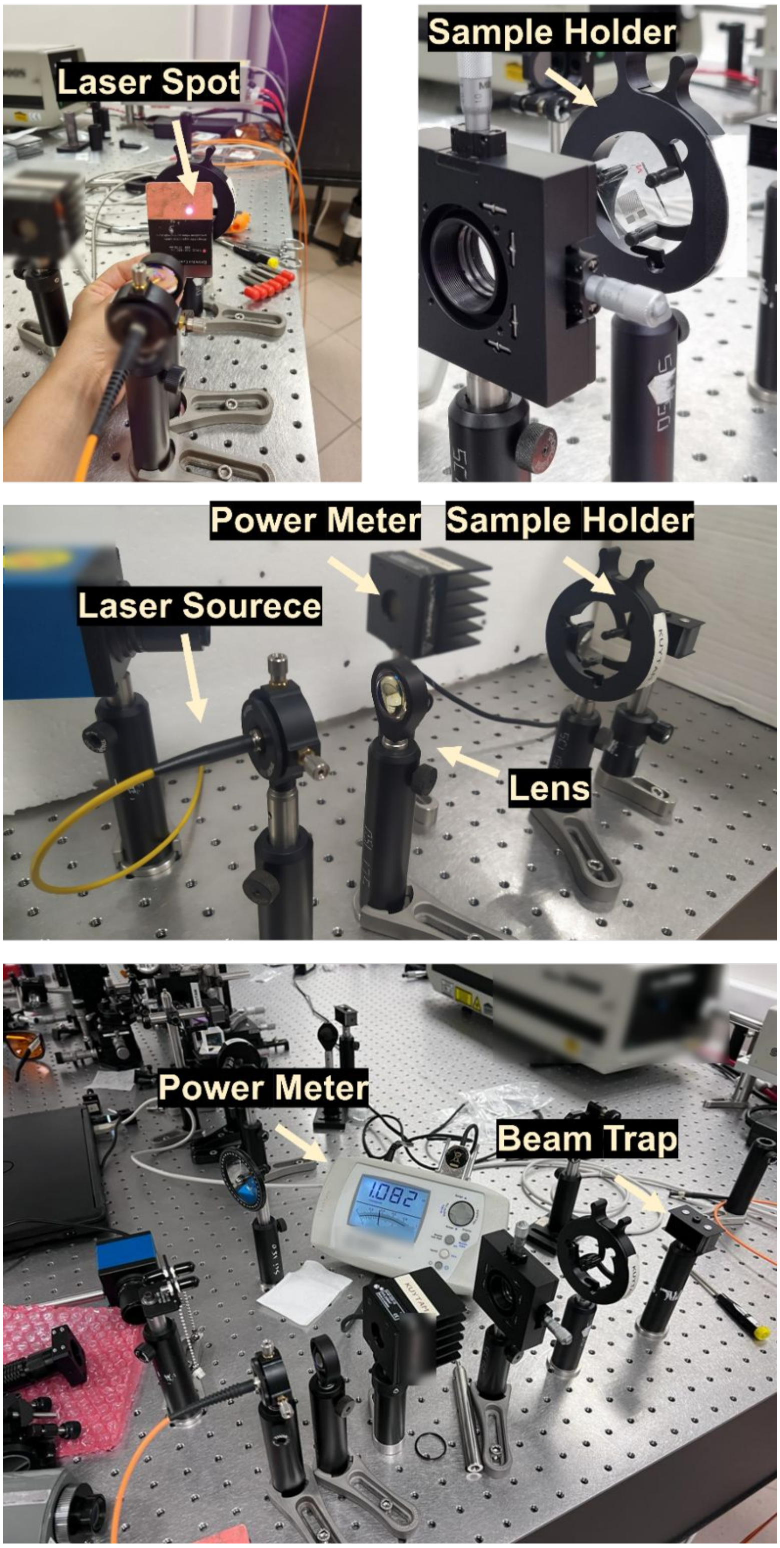

Figure S4. NIR laser setup

4. Structural characterization of NIR exposed samples

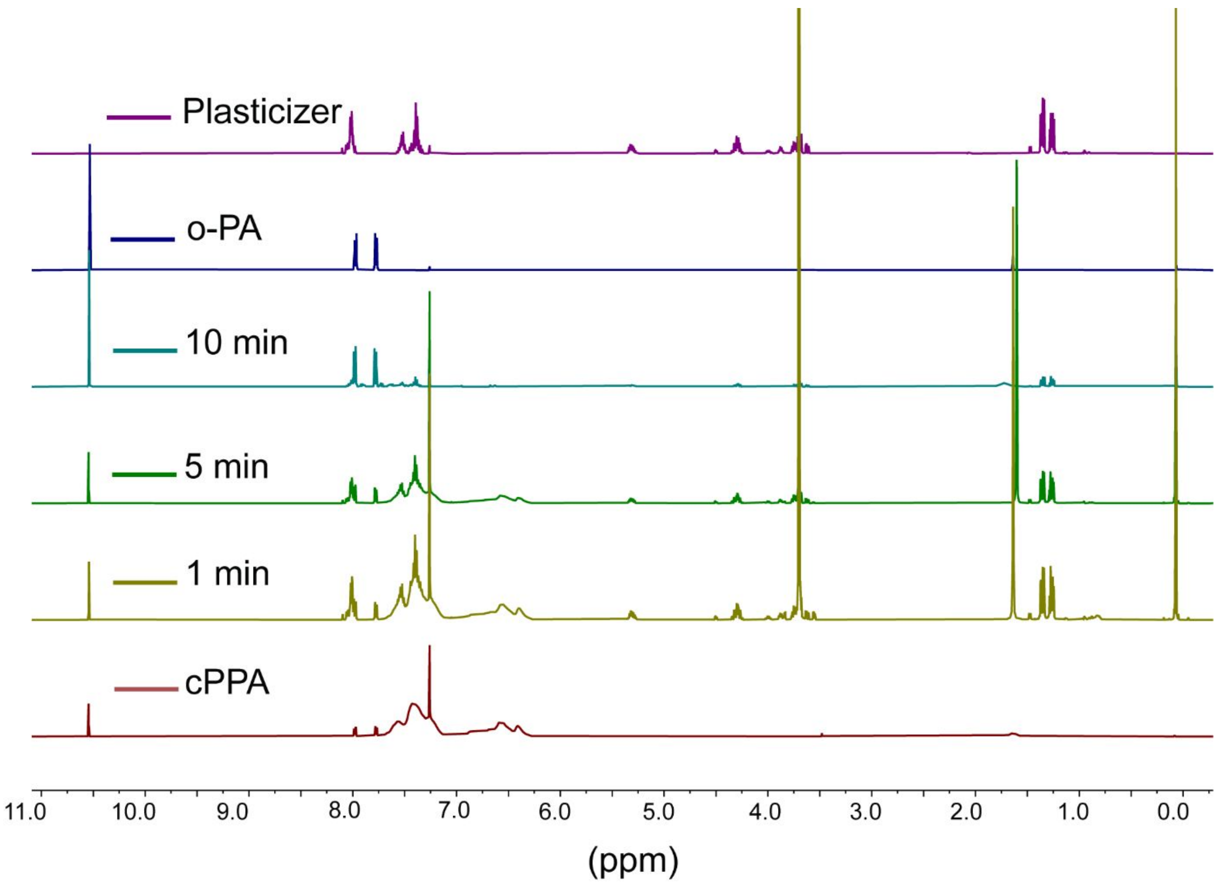

Figure S5. ^{1}H NMR spectra of the exposed, non-exposed, monomer and plasticizer in $CDCl_3$.

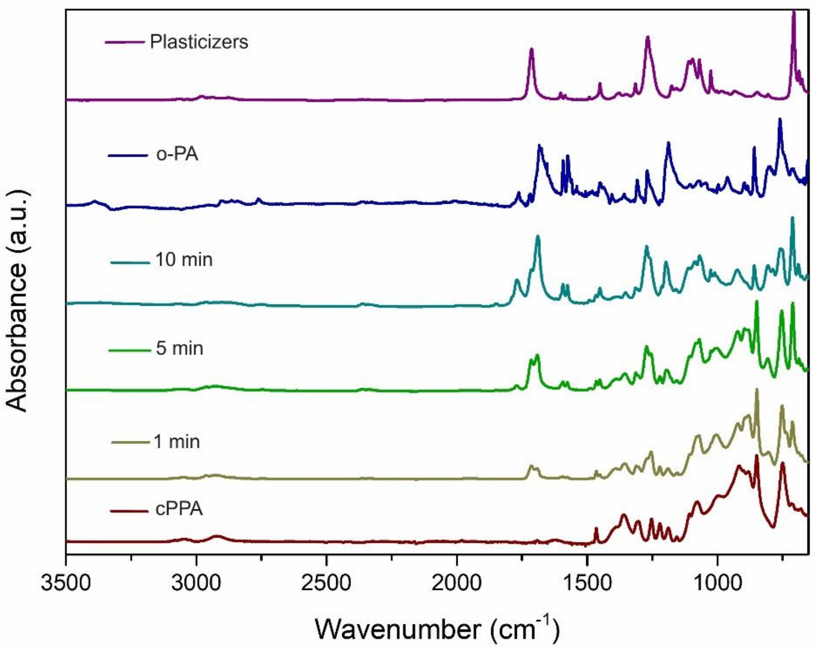

Figure S6. FTIR spectra of the exposed (1 min-green/olive) (5 min-green) (10 min-green/forest), non-exposed (red), monomer (navy) and plasticizer (purple).

5. Optical images of exposed and non-exposed samples

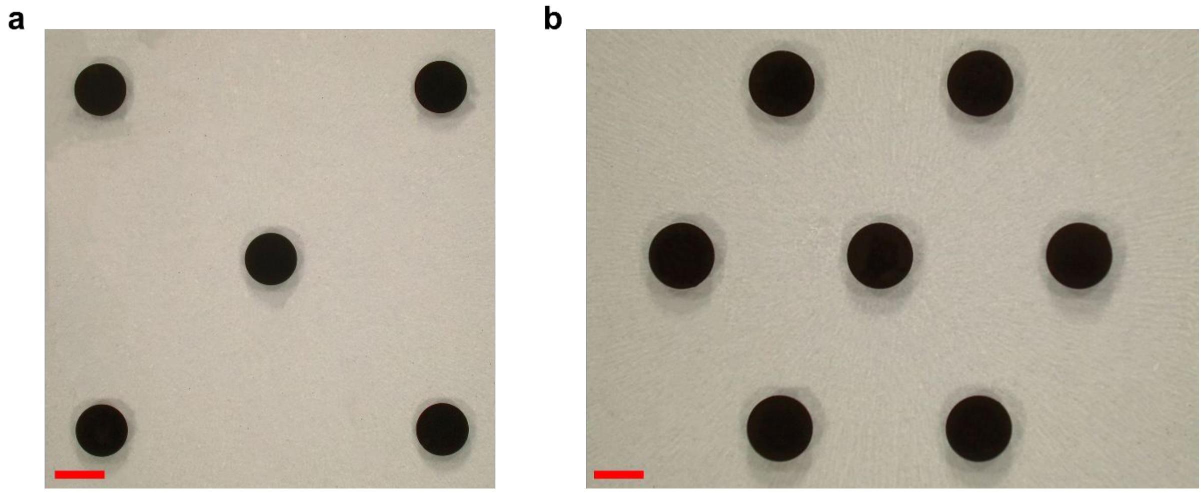

Figure S7. Fabricated Mo based circle shape specimen fabricated on glass substrate using e-beam evaporation, a) 5 separated Mo circle, b) 7 separated Mo circle. (scale bar=2mm)

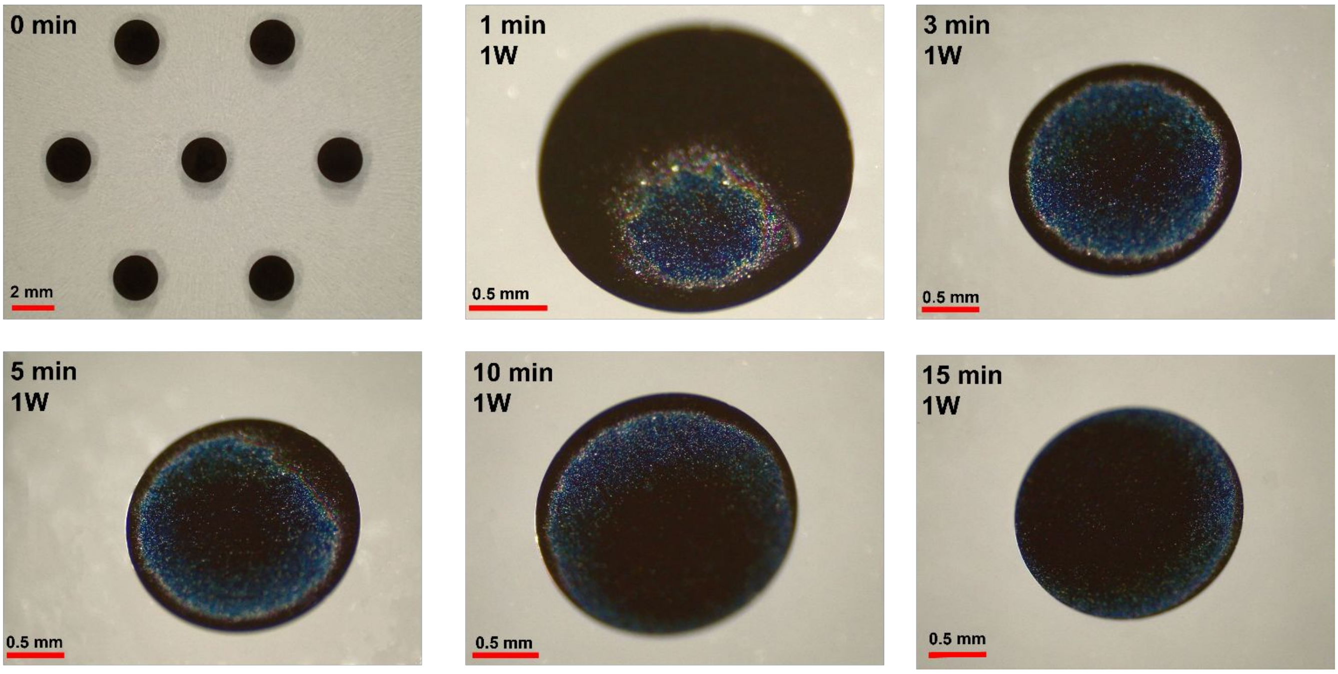

Figure S8. Optical microscopy images of NIR exposed cPPA film coated on Mo.

6. ^{1}H NMR characterization of thermally degraded cPPA film

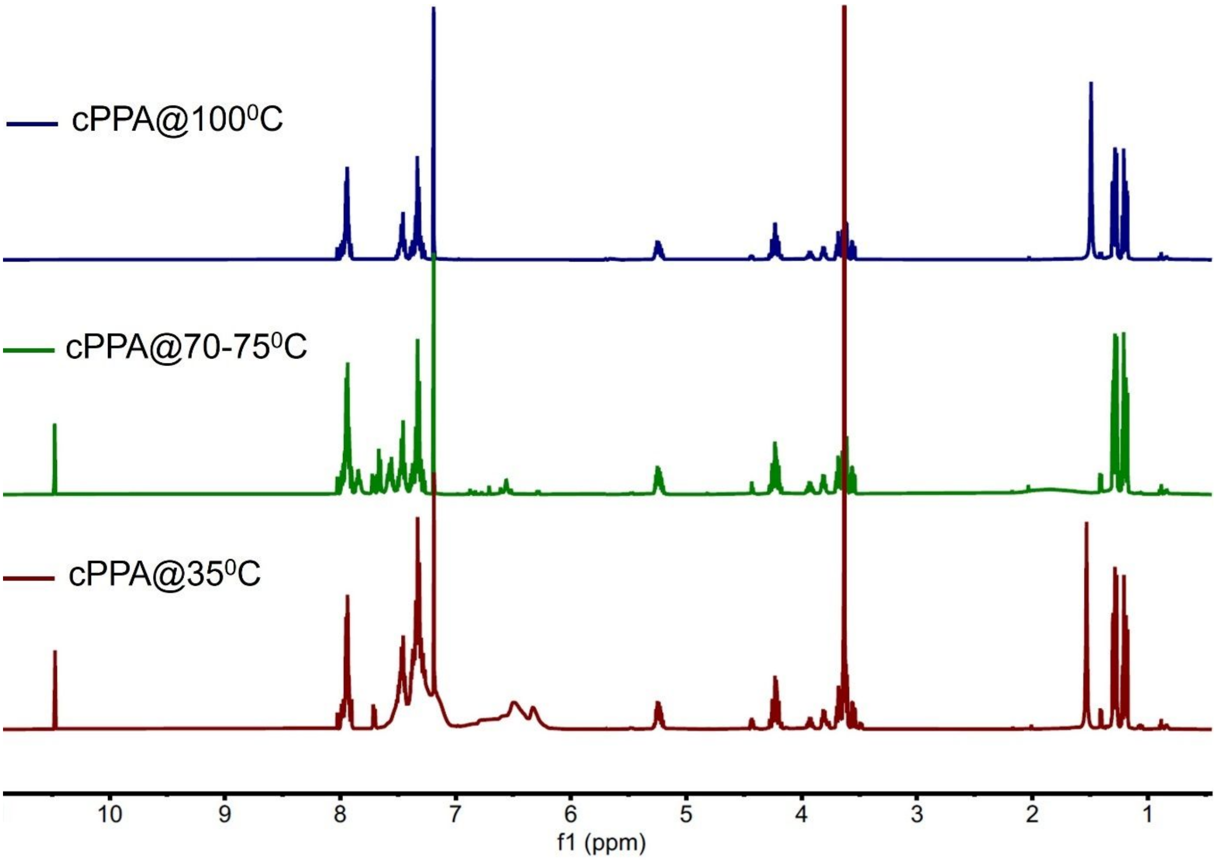

Figure S9. Degradation of cPPA using thermal treatment in various temperature range.

7. Profilometry characterization of the samples

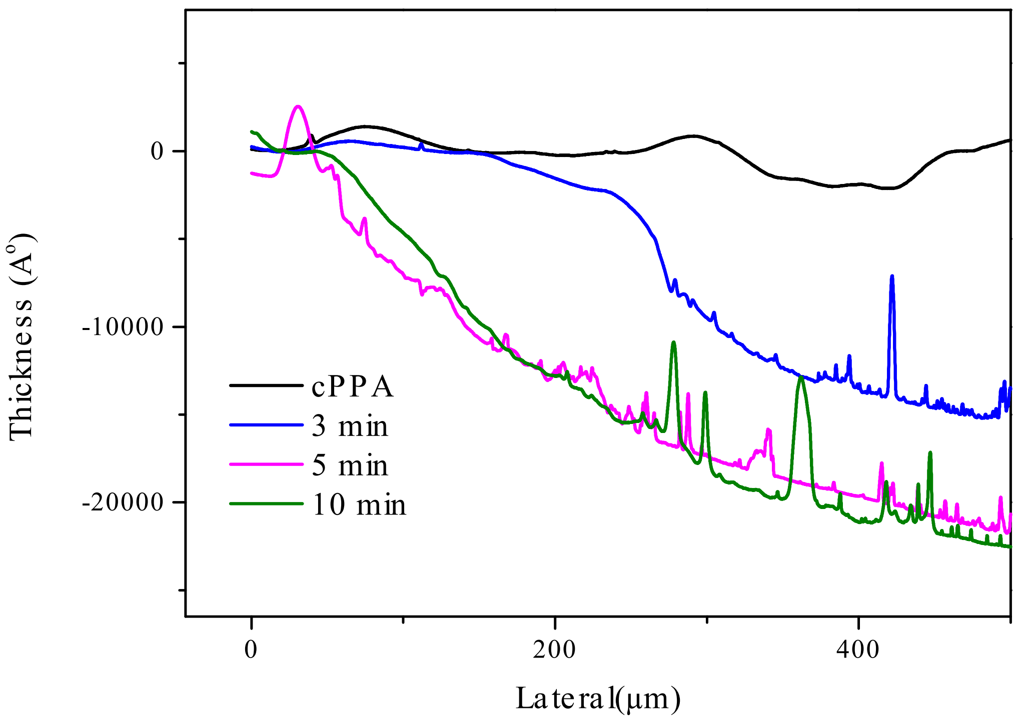

Figure S10. Profilometer results of the NIR exposed samples.

8. Contact angle characterizations

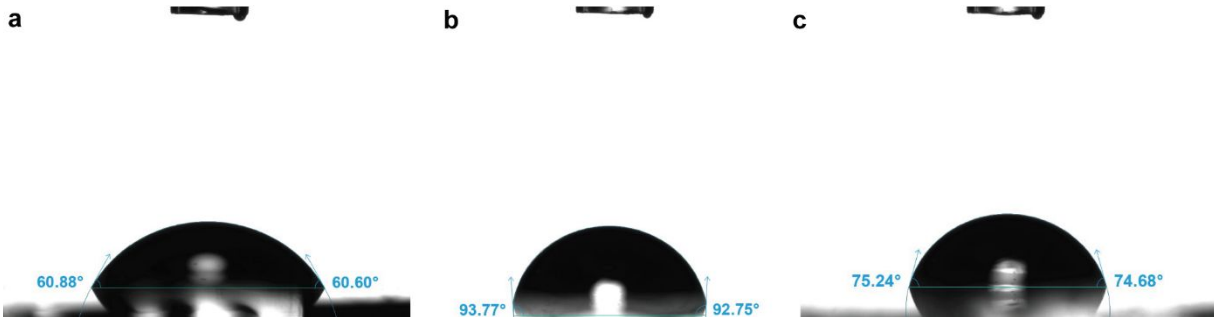

Figure S11. Contact angle measurements of a) bare Mo, b) bare cPPA and c) NIR exposed cPPA sample on Mo.

9. Mo-based electrodes

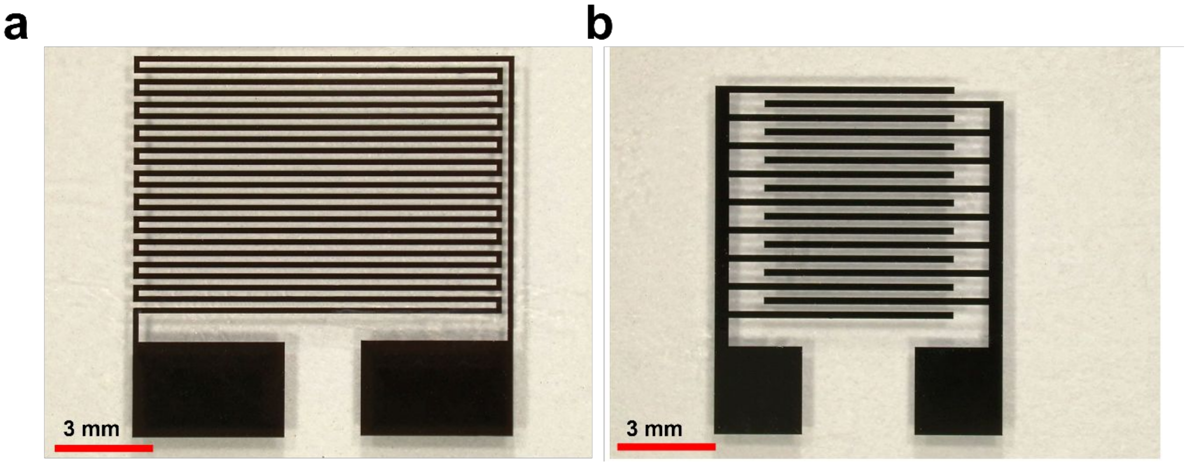

Figure S12. Mo based a) resistor and b) interdigitated electrodes

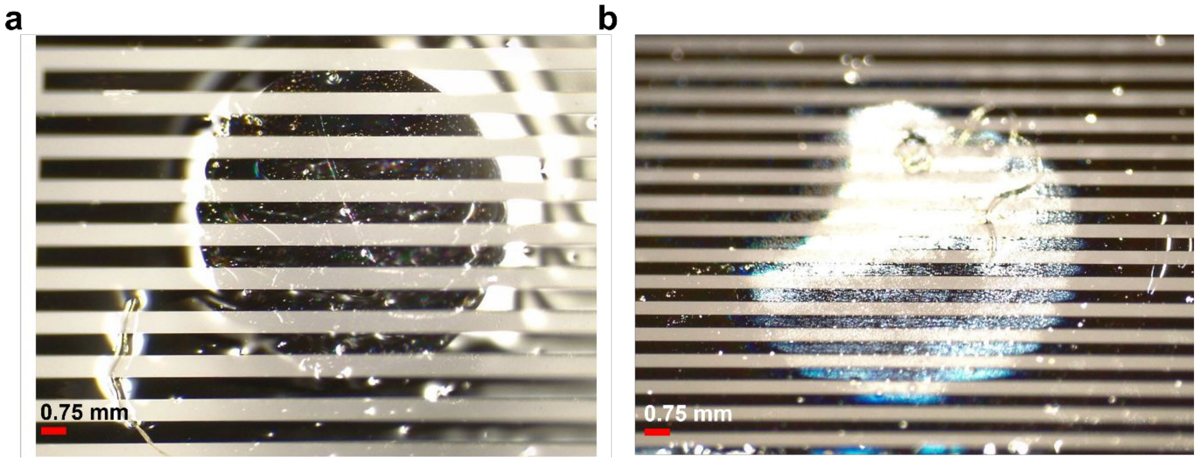

Figure S13. Degradation of cPPA on electrodes after NIR exposure, a) interdigitated Mo electrode, b) Mo resistor.

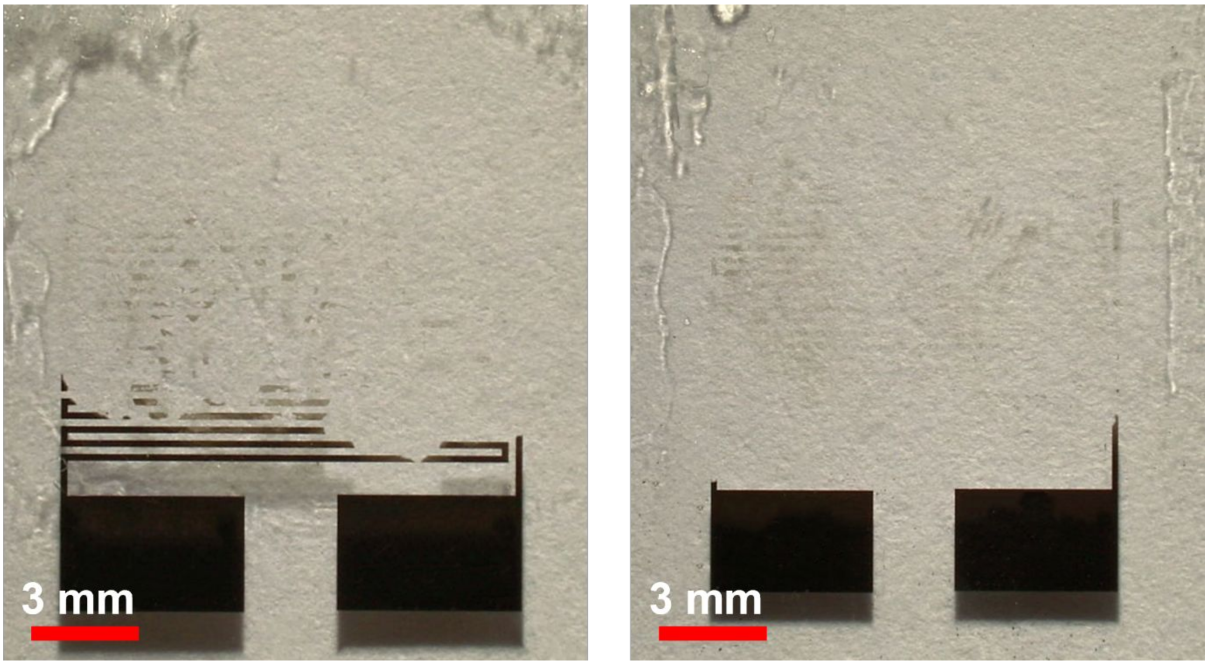

Figure S14. Dissolution of Mo electrode after basic treatment

10. Capacitance data of Mo based capacitors

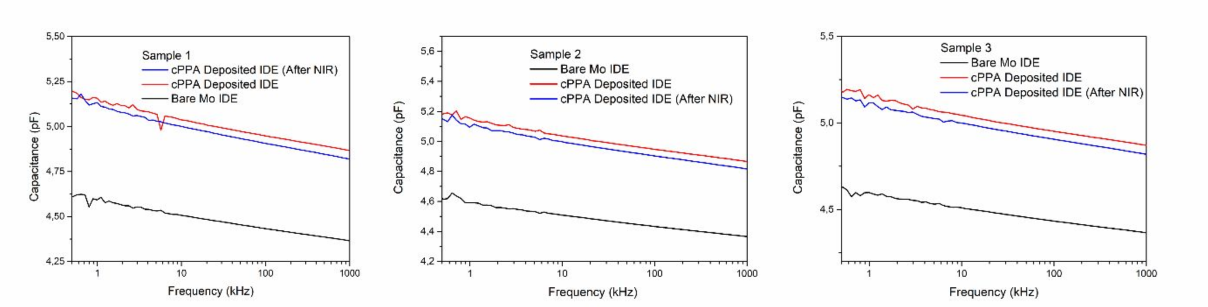

Figure S15. Capacitance measurement of 3 different IDEs electrode

11. Resistance data of Mo based resistors

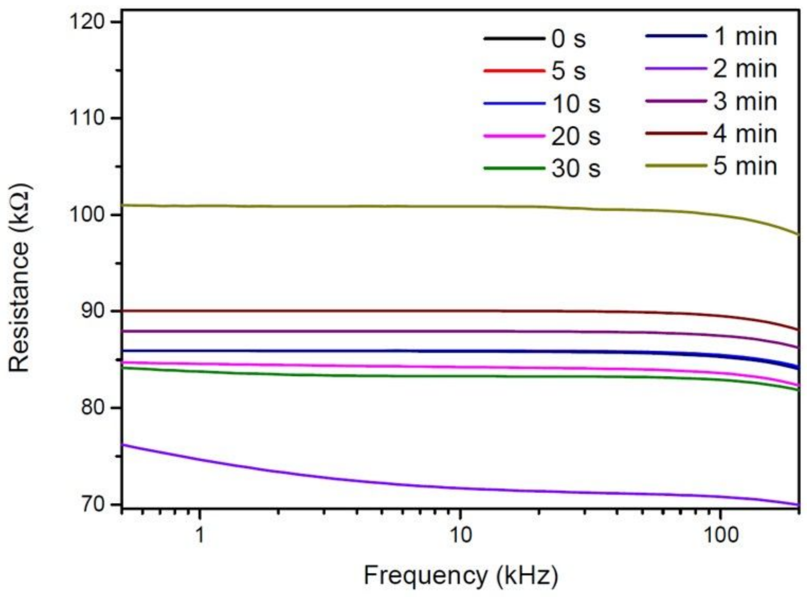

Figure S16. The resistivity change data of NIR exposed cPPA coated Mo resistors after basic PBS treatment up to 5 minutes.

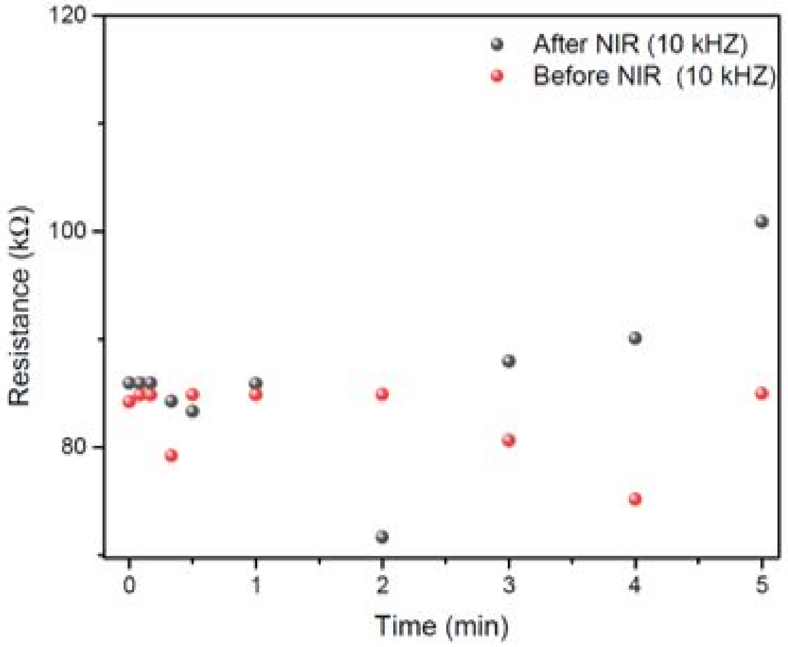

Figure S17. The resistivity change data of NIR exposed cPPA coated Mo resistors at 10 kHz as a function of time between 0 to 5 min after basic PBS treatment

12. Mechanical analysis of the samples

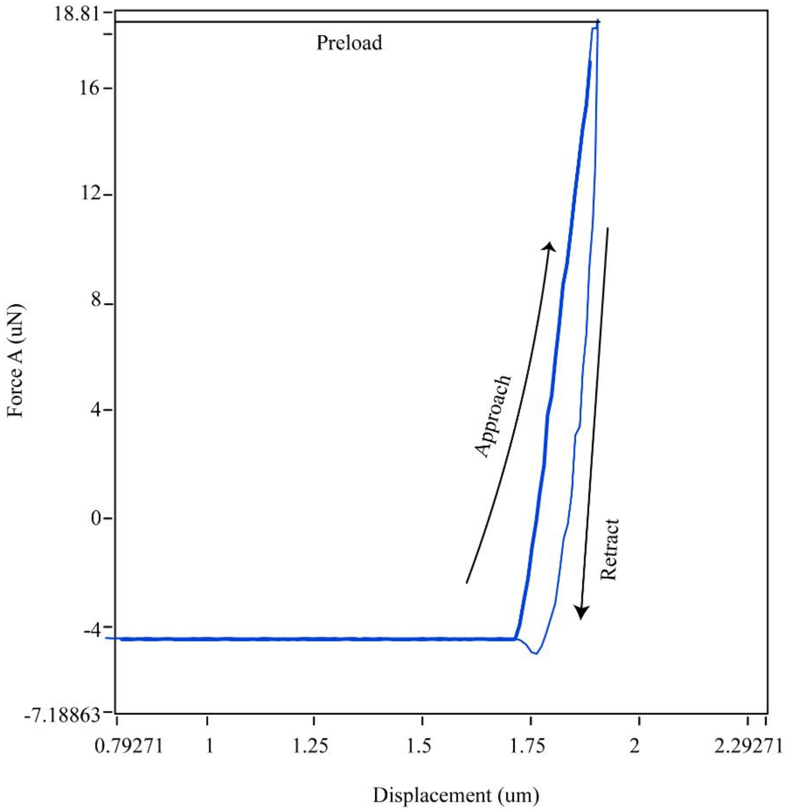

Figure S18. Force-displacement curve during a typically loading-unloading dynamic mechanical analysis

Figure illustrates a schematic force-displacement curve during a typically loading-unloading dynamic mechanical analysis. The deviation of the unloading curve from the loading curve can be attributed to the presence of plastic deformation. In the Oliver-Pharr method, the contact stiffness, denoted as S, is determined by evaluating the slop of the

unloading segment of a dynamic mechanical analysis curve when the maximum load, F_{max}, is reached. Oliver and Pharr suggested that if the tip-shape function[1], denoted as A_c is a function of contact depth (h_c), A_c can be determined by measuring the contact depth h_c when the indenter is at full load. For a spherical tip with a radius of R_2, the contact depth hc is calculated as hc ≈ a^2 / (2 * R_2), and therefore A_c is approximately proportional to h_c, expressed as A_c ≈ 2πR2 * h_c, or equivalently, A_c ~ h_c.

The following formula was utilized to compute the contact depth h_c from the force-displacement curve.

$$h_c = h_{max} - \varepsilon \frac{F_{max}}{S} \quad (1)$$

In this context, hmax represents the complete displacement of the indenter at the point of reaching the maximum force, Fmax.The variable ε is a constant specific to a particular indenter tip, and for spherical indenter tip ε = 0.75. After the calculation of contact depth, reduced modulus was determined using the following formula:

$$S = \frac{2}{\sqrt{\pi}} E_r \sqrt{A_c} \quad (2)$$

The reduced modulus, denoted as E_r, takes into consideration the deformation of both the indenter, characterized by its elastic modulus (Ei) and Poisson's ratio (νi), and the sample, characterized by its elastic modulus (Es) and Poisson's ratio (νs). The formula for calculating E_r is as follows:

$$\frac{1}{E_r} = \frac{1 - V_s^2}{E_s} - \frac{1 - v_i^2}{E_i} \quad (3)$$

Utilizing equation 3, elastic modulus values were computed.

References

1. White, C. C., VanLandingham, M. R., Drzal, P. L., Chang, N. K., & Chang, S. H. (2005). Viscoelastic characterization of polymers using instrumented indentation. II. Dynamic testing. Journal of Polymer Science Part B: Polymer Physics, 43(14), 1812-1824.
